# Diseases of the Digestive Organs

**Published:** 1905-04-15

**Authors:** 


### Progress in Medicine and Surgery.

### DISEASES OF THE DIGESTIVE ORGANS.

(*Continued from page* 33.)

**Jaundice.**—W. H. Porter,[12] in speaking of obstructive jaundice, lays down that there are two forms, one acting outside the liver and one within the lobules. The latter can only be treated medically, the other is amenable to surgery. Medical treatment should commence with such a diet as the system can digest and oxidise. Thus skim milk is to be followed by whole milk, meat broths, raw eggs, and, later on, lean meat. Last of all bread may be given as the digestion improves. In this way we must aim at preventing the poisons due to abnormal fermentation. An action of the bowels two or three times daily to carry off toxins should be insured. At the same time to affect directly the liver cells he gives perchloride or subchloride of mercury with ipecacuanha or arsenic. J. F. H. Broadbent[13] reports a case of toxæmic jaundice in a girl of 17 years fatal in five weeks. The liver was rather small and showed red hæmorrhagic layers alternating with yellow fatty layers. The fatty change was most marked around the portal vein. The pyrexia and other symptoms pointed to the absorption of some toxin from the intestines and presented many analogies to acute yellow atrophy. He also discussed four cases of interstitial fibrosis which ended in pyrexia, jaundice, and coma, and thinks that many obscure cases of cirrhosis are due to cicatrisation of the liver after a general toxæmia, as in fevers, the liver being infected through the hepatic artery, though the fibrosis is started around the portal canals in some cases, and in others within the lobules. Poynton remarked that rheumatism and endocarditis rarely caused cirrhosis of the liver. Infective jaundice was the subject of a series of papers at the Oxford meeting. Epidemics occur in various parts of the world. In Asia Minor and Egypt it has existed for many years, and the question of its identity with Weil's disease was discussed. Sandwith[14] thinks it may be carried by insects. The mortality in Egypt is about 30 per cent., and it has been suggested that this is really a mild form of yellow fever, but the temperature differs, and albuminuria is not so marked a feature. Anderson reported epidemics from India, and Mathias from South Africa, where 5,600 cases of jaundice were reported during the war. Here the mortality was very low, as in Weil's disease. Jaeger, of Ulm,[15] found in cases of the latter affection an organism, the B. proteus fluorescens, which also existed in the water of the Danube. Brüning found recently the same bacillus in an infant who died from jaundice with fever, diarrhœa, and albuminuria. It was noticeable that the serum had marked agglutinating power over the typhoid bacillus. This relation has been noted before, and it will be interesting to note whether this bacillus is often found in infectious jaundice.

**Cirrhosis.**—H. Richardson[16] remarks that atrophic cirrhosis of the liver is associated with interstitial nephritis, cardiac failure, and arterial cirrhosis and other diseases. Its cause is, (1) the absorption of toxins from the intestines; (2) failure to excrete them, due to some hindrance to the outflow of bile. Syphilis and malaria also produce toxins and bilirubin in excess, which act in the same way, and various toxins in the blood hinder the outflow of bile by increasing its viscosity. Again, gall-stones are accompanied with a deficiency of bile acids compared to the amount of cholesterin. The treatment of cirrhosis, then, depends (1) on the removal of the cause of the toxæmia, such as the special dyspepsia present; (2) on the stimulation of the liver to increase the oxidation and excretion of toxins in the bile. The only cholagogues known are bile salts. Hence we should give sodium glycocholate with small doses of mercury, while the fluidity of the bile should be increased by alkaline mineral waters, and especially by salicylate of soda. In cases associated with nephritis, biniodide of mercury with iodide of potash may be helpful.

**Cholecystitis.**—A. M. Pond[17] records a case of biliary colic due to the presence of ascaris lumbricoides in the duct. Martens has collected 48 cases, in two of which the cause was discovered during life. Pond's patient was a navvy, aged 34, suffering from colic, jaundice, enlarged tender liver, and tumour in the region of gall-bladder. Biliary pigments and acids were present in the urine. After repeated doses of calomel and magnesia a round worm was passed, operation was postponed, and the patient recovered. The worm in such cases causes stagnation of bile and this leads to bacterial invasion and inflammation of the ducts. He quotes Kehr to the effect that the pain in colic is due, not to the stone, but to the transudation of fluid into the bile channels, caused by the inflammatory changes which follow biliary obstruction. T. G. Sheldon[18] insists that acute cholecystitis occurs not only as a result of gall-stones and after fevers, but as a primary affection, and reports four cases of his own in which this condition was present. The causation is unknown. It may be due to the colon or typhoid bacillus. If they lodge in the cystic duct and cause obstruction, inflammation and gangrene will quickly follow with peritonitis. There is usually in these cases a rigor, sudden violent pain, fever, local tenderness, and usually vomiting, but jaundice is not found in uncomplicated attacks. Peritonitis quickly follows in severe ones. The writer advises early operation. Except where extensive gangrene is present, a cholecystotomy is preferable.

**Constipation.**—G. Herschell [19] recommends injections of olive oil for obstinate cases and also in membranous colitis, where it also reduces the amount of mucus. The injections are specially useful in chronic colitis, in constipation due to spasm of the bowel in neurasthenics, and during the electrical treatment of atony of the bowel. Three to ten ounces of warm oil should be injected at bedtime and retained. A Higginson's syringe causes spasm, so that a douche with a wide nozzle should be used, and the oil given every night for two or three weeks, next on alternate nights, and finally only on those days when the bowels fail to act.

**Ulcerative Colitis.**—A. Garrick Wilson [20] reports a case in a male aged 51. There had been intermittent diarrhœa for two years, worse for some months and extreme anæmia. The temperature was subnormal, the motions were painless, five to ten daily, chiefly blood with some mucus and small pellets of fæcal matter. Ulceration could be felt in the rectum, and there was constant severe pain in the lower part of the abdomen and tenderness in the iliac fossæ. Salol, opium, bismuth, and injections of laudanum and of 10 grains of nitrate of silver in half-a-pint of water were given, but the patient was too far gone to stand colotomy. At the post mortem multiple perforations were found in the colon. There were numerous serpiginous ulcers with undermined edges increasing in number towards the rectum. The remaining islets of mucous membrane were swollen and showed papillomatous outgrowths. There was no evidence of typhoid, and as the patient had never been out of Yorkshire, and for other reasons, dysentery was negatived. Chronic granular kidney was present, and there was a history of constipation with occasional diarrhœa for some years. Argyrol injections have been found useful in a similar case, but colotomy seems advisable when it is possible. The bacillus coli and another large bacillus were found in the stools.

**Dysentery.**—While Shiga's bacillary type occurs both in temperate and warm climates, the amœbic form arises usually after or during a stay in hot climates. Dopter [22] brought forward five cases of the latter in soldiers who had never been abroad, but who had barrack comrades infected in the tropics. Thus in one man the amœba was present and was conveyed by injection to a cat with a fatal result. Shiga's bacillus could not be found and agglutination tests for it were negative. This tends to show that the amœbic form may arise and spread in this country. The disease begins slowly, may become chronic with relapses, and may cause liver abscess. The bacillary form begins suddenly, and ends in complete recovery or death in a few weeks. Relapses are rare and the liver is never affected. Moreover, it may be cut short by injections of serum from an immunised horse. However, other writers consider the question is more complex, that there are "acid" and "alkaline" types of bacillary dysentery, and that serum should be prepared from mixed cultures. Morgenroth found in China cases of liver abscess, though no amœba was in the stools. In four cases he found Flexner's bacillus and in others Shiga's. Castellani [22] in Ceylon found also a spirillum, a bacillus like B. typhosus, and a para-dysenteric bacillus closely allied to Shiga's. Todd shows that the toxin of the bacillus isolated in outbreaks in English asylums is neutralised by antitoxin prepared from Shiga's. Hence the bacilli are the same or closely allied.

[12] Med. Rec., Sept. 24. [13] Lancet, Oct. 22. [14] Brit. Med. Jour., Sept. 17. [15] Lancet, Nov. 22. [16] Med. Rec., Oct. 8. [17] Amer. Jour. Med. Sci., Sept. [18] Ibid. [19] Lancet, Oct. 1. [20] Ibid., Oct. 29. [21] Ibid., Dec. 3. [22] Jour. of Hygiene, p. 495, 1904.

